# Traumatic Spinal Cord Injury among Patients Admitted to the Spine Unit in a Tertiary Care Centre

**DOI:** 10.31729/jnma.8292

**Published:** 2023-10-31

**Authors:** Samaj Gautam, Badri Rijal, Laxmi Kanta Sharma

**Affiliations:** 1 Department of Orthopaedics, National Trauma Center, Mahankal, Kathmandu, Nepal

**Keywords:** *falls*, *prevalence*, *spinal cord injuries*

## Abstract

**Introduction:**

Spinal cord injury usually results in disabling conditions. The incidence of spinal trauma is region-specific due to unique geography and demography. The epidemiology of spinal trauma changes with economic and social factors even in different periods. The aim of this study was to find out the prevalence of traumatic spinal cord injury among patients admitted to the Spine Unit in a tertiary care centre.

**Methods:**

A descriptive cross-sectional study was done in a tertiary care centre among patients admitted to the Spine Unit from 1 January 2022 to 31 December 2022 after receiving ethical approval from the Institutional Review Committee. Demographic details, mode of injury, level of injuries, neurological grading at the time of admission using American Spinal Injury Association grading, management methods, and complication if any were recorded. A convenience sampling method was used. The point estimate was calculated at a 95% Confidence Interval.

**Results:**

Out of 465 patients, the prevalence of traumatic spinal cord injury was 316 (67.95%) (63.7272.20, 95% Confidence Interval). A total of 243 (76.89%) cases were due to falls. The mean age of patients was 43.13±16.55 years.

**Conclusions:**

The prevalence of traumatic spinal cord injury patients was lower than the other studies done in similar settings.

## INTRODUCTION

Traumatic spine injury is the major cause of morbidity and mortality in the world.^1^ The impact of spinal injuries is high in terms of cost and recovery time.^2^ The cases of spinal injuries reported to the tertiary care centre of our country are increasing nowadays but proper data recording is very rare.^1^ Proper recording is necessary to develop national policy and guidelines for its management.

The incidence of spinal trauma is region-specific due to unique geography and demographics.^3^ The epidemiology of spinal trauma changes with economic and social factors even in different periods.^4^ The incidence and prevalence of traumatic spinal injury are different between our region and Western countries thus management policy needs to be tailored according to region.^5^ A proper understanding and injury-related trends in the injured population helps to direct and formulate health-related plans and policies.^3^

The aim of this study was to find out the prevalence of traumatic spinal cord injury among patients admitted to the Spine Unit in a tertiary care centre.

## METHODS

This descriptive cross-sectional study was conducted among patients visiting the Spine Unit at the National Trauma Center, Mahankal, Kathmandu, Nepal from 1 January 2022 to 31 December 2022. Ethical approval was taken from the Institutional Review Committee (Reference number: 915/2079/80). Data were collected from 2 June 2023 to 11 June 2023. Spinal injuries from C1 to sacrum, of any morphology in any age group during the study period were included. Pathological fractures and multiple fractures (spine with other orthopaedic fractures) were excluded. A convenience sampling method was used. The sample size was calculated using the following formula:


$$\begin{array}{ll} \text{n} & ={\text{Z}}^{2}\times \frac{\text{p}\times \text{q}}{{\text{e}}^{\text{2}}}\\ & =1.96^{2}\times \frac{0.78\times 0.21}{0.04^{2}}\\ & =406 \end{array}$$

Where,

n = minimum required sample size
Z = 1.96 at 95% Confidence Interval (CI)
p = prevalence of traumatic spinal cord injury taken from previous study, 78.46%^6^
q = 1-p
e = margin of error, 4%

The calculated sample size was 406. After adjusting 10% for the non-response rate, the calculated sample size was 451. However, 465 patients were included.

Structured coded data and non-structured narrative data were taken from electronic hospital data. Nonelectronic hospital records [patient files, scanned documents, and images (X-rays, CT scan, and MRI)] was evaluated to complete the missing data from electronic hospital record. Demographics details, mode of injury, aetiology of injury, neurological levels at the time of admission [American Spinal Injury Association (ASIA) grading], and management methods (conservative versus surgical), and complications if any were recorded. The international spinal cord injury core data set (version 3) was used for data collection to standardize the collection and report the information on spinal cord injury patients.^7,8^

The data collected were entered in Microsoft Excel 2010 and analyzed using IBM SPSS Statistics version 25.0. The point estimate was calculated at a 95% CI.

## RESULTS

Out of 465 patients, the prevalence of traumatic spinal cord injury patients was 316 (67.95%) (63.72-72.20, 95% CI). A total of 210 (66.46%) were male with a male-to-female ratio of 1.98. The mean age of patients was 43.13±16.55 years (Table 1).

**Table 1:** Demographic profile of patients with traumatic spinal cord injuries (n= 316).

Characteristics	n (%)
**Gender**	
Male	210 (66.46)
Female	106 (33.54)
**Mode of injury**	
Fall injury	243 (76.89)
Road traffic accidents	44 (13.92)
Blunt trauma	26 (8.22)
Suicidal attempts	3 (0.94)
**Regions involved**	
Cervical	88 (27.84)
C6/C7	50 (56.81)
Dorsal	77 (24.36)
Lumbar	141 (46.51)
Dorso-lumbar junction (D10-L2)	132 (60.53)
Sacrum	4 (1.26)

At the time of admission, 115 (36.39%) had ASIA E and 103 (32.59%) had ASIA A neurological grading. At the time of discharge, there were 125 (39.55%) cases of ASIA E grade at the time of discharge (Table 2).

**Table 2:** Neurological grading of patients with spinal cord injuries (n = 316).

Grade	At admission n (%)	At discharge n (%)
A	103 (32.59)	98 (31.01)
B	27 (9.17)	27 (9.71)
C	28 (8.86)	20 (6.32)
D	43 (13.60)	51 (16.13)
E	115 (36.39)	125 (39.55)

A total of 196 (62.02%) patients were surgically treated whereas 120 (37.97%) were treated conservatively. Region-wise, 64 (72.72%) cervical cases and 132 (60.55%) dorso-lumbar cases were treated surgically (Table 3).

**Table 3:** Management of patients with spinal cord injuries (n= 316).

Characteristics	Cervical	Dorso-lumbar
Conservative	22 (28.28)	86 (39.45)
Surgery	64 (72.72)	132 (60.55)
Anterior	29 (32.95)	-
Anterior cervical discectomy and fusion	20 (22.72)	-
Anterior cervical corpectomy and fusion	9 (10.22)	-
Posterior	35 (39.77)	132 (60.55)
Open	35 (39.77)	108 (49.54)
Minimally invasive spinal surgery	-	24 (11.21)

The majority of 102 (52.04%) cases underwent surgery after 7 days from the time of hospital admission. A total of 82 (41.83%) underwent surgery within 3 to 7 days from the day of admission whereas only 12 (6.10%) cases had surgery within 3 days from admission. The mean hospital stay was 15.25±14.03 days.

A total of 40 (12.65%) cases needed intensive care unit admission. There was no in-hospital mortality. A total of 12 (6.10%) cases developed surgical site infections out of which 2 (16.67%) deep surgical site infections were treated with debridement and intravenous antibiotics and other were treated conservatively. There were 4 (1.26%) grade 3 bed sore and 4 (1.26%) grade 2 bed sore in ASIA A patients who were treated by a plastic surgeon.

## DISCUSSION

The prevalence of traumatic spinal cord injury in this study was 67.95% which was slightly lower (78.46%) than the study done in a similar setting.^6^ The incidence and prevalence of traumatic spinal injury are different between our region and Western countries thus management policy needs to be tailored according to region.^5^

The mean age of patients in our study was 43.13±16.55 years (10-83 years) which is similar to 44.06 years as shown by another study done in the western part of the country.^6,9^ One systemic review showed the mean age to be 30.7-48.5 years in developed countries and less than 30 years in developing countries.^10^ The most injured age group was 46-60 years according to a study done.^11,12^ Our study showed maximum patients in the age group of 31-45 years and 46-60 years. These studies show that the most vulnerable age for spinal injury is the productive age group thus creating a direct impact on the financial aspect of the family. People from this age group usually go outside for work thus risking themselves for injury.

Generally falls are the most common cause of spine trauma in developing countries whereas road traffic accidents are the most common cause in developed countries.^10,13^ Active males are more prone to injury in every country.^5,10,14^ Our study also concluded that 76.89% of cases were due to falls and 13.92% due to road traffic accidents. A total of 66.46% of cases were males in our study with a male-female ratio of 1.98. Being an agricultural country most male people from our country go to fields and jungle for work where they get injured.

The Lumbar region was the most common 46.51% involved region in our study followed by cervical (27.84%) and dorsal (24.36%). Studies done in similar settings also showed the lumbar region to be commonly involved,^9^ whereas other studies showed the cervical to be the most common.^6,10,14^ In our study at the time of admission, 115 (36.39%) had ASIA E and 103 (32.59% ) had ASIA A neurological grading. At the time of discharge, 46 (22.88%) patients out of 201 had neurological improvement of one grade. Most of the other studies also showed that the maximum number of patients had ASIA E neurological grading.^4,11,15^ There was one grade of neurological improvement in 21.39% of our cases which was similar to other studies.^4,11^ Studies have shown earlier the treatment, the sooner and more the recovery of neurology.^16,17^

Generally unstable fractures or fractures associated with neurological injury are treated surgically. In our study 120 (37.97%) cases were treated conservatively with analgesics, braces, and physiotherapy. A total of 196 (62.02%) cases were treated surgically. Another study done in similar settings also showed 60%^6^ and 58.8%^9^ of cases treated with various surgical methods. In our study among the surgically treated cervical injuries posterior surgery was done in 35 cases (55.85%) and anterior surgery in 29 (45.15%) cases. In the dorso-lumbar region, all the surgery were done from the posterior approach.

The financial constraints, the topographical configuration, and the transport facilities of our country are not so satisfactory for these patients to reach the hospital in time. Moreover, the awareness of the illness is also a limiting factor. Usually, these patients land at the hospital late, and the high cost of treatment further delays the management. Our study showed 102 (52.04%) cases underwent surgery after seven days of admission, 82 (41.83%) cases after three to seven days, and only 12 (6.1%) cases within three days. Studies have shown earlier that surgery sooner and more the neurological recovery and it was a similar finding in our study. Another study done in a similar setting showed only 38.35% of cases got admitted to the hospital within three days and the mean surgery time was 11-15 days from the day of admission.^12^ Another study showed that 50% of cases got admitted to the hospital after 3-7 days of injury and 53.3% of cases underwent surgery in 8-30 days.^15^

The mean duration of hospital stay was 15.25±14.03 days (range 1-80 days) in our study. Our hospital has its own rehabilitation and physiotherapy service so that patients stay longer in the hospital. A similar other study showed mean hospital stay to be 31±13 days,^12^ 22.4±2.2,^4^ and 18.76 days.^9^ Besides neurological recovery, early surgery is recommended to reduce in-hospital complications and morbidities.^16,17^ 2.53% of bed sores were seen in ASIA A patients who were treated by a plastic surgeon. One study showed a 2.02% mortality rate,^4^ whereas we did not have any inhospital mortality.

Our study has certain limitations. This was a singlecentred descriptive cross-sectional study with a small number of patients. The patients who were discharged or left the emergency without treatment were missed from the study. Primary treatment at the site of injury and methods of transfer could not be traced. This study does not follow the patient after discharge so functional and radiological outcomes could not be evaluated. The rehabilitation and vocational integration of these patients have not been studied.

## CONCLUSIONS

The prevalence of traumatic spinal injury patients was lower than other studies done in similar settings.
